# Autonomous Underwater Vehicles: Identifying Critical Issues and Future Perspectives in Image Acquisition

**DOI:** 10.3390/s23104986

**Published:** 2023-05-22

**Authors:** Alberto Monterroso Muñoz, Maria-Jose Moron-Fernández, Daniel Cascado-Caballero, Fernando Diaz-del-Rio, Pedro Real

**Affiliations:** 1 Department of Applied Mathematics I, Universidad de Sevilla, 41012 Sevilla, Spain; real@us.es; 2 Department of Computer Architecture and Technology, Universidad de Sevilla, 41012 Sevilla, Spain; mjmoron@us.es (M.-J.M.-F.); danicas@us.es (D.C.-C.)

**Keywords:** underwater image, sensing for autonomous underwater vehicles, optical imaging technologies, image acquisition, image processing methods

## Abstract

Underwater imaging has been present for many decades due to its relevance in vision and navigation systems. In recent years, advances in robotics have led to the availability of autonomous or unmanned underwater vehicles (AUVs, UUVs). Despite the rapid development of new studies and promising algorithms in this field, there is currently a lack of research toward standardized, general-approach proposals. This issue has been stated in the literature as a limiting factor to be addressed in the future. The key starting point of this work is to identify a synergistic effect between professional photography and scientific fields by analyzing image acquisition issues. Subsequently, we discuss underwater image enhancement and quality assessment, image mosaicking and algorithmic concerns as the last processing step. In this line, statistics about 120 AUV articles fro recent decades have been analyzed, with a special focus on state-of-the-art papers from recent years. Therefore, the aim of this paper is to identify critical issues in autonomous underwater vehicles encompassing the entire process, starting from optical issues in image sensing and ending with some issues related to algorithmic processing. In addition, a global underwater workflow is proposed, extracting future requirements, outcome effects and new perspectives in this context.

## 1. Introduction

Underwater environment sensing has been a challenging field since its inception. The evolution of autonomous underwater vehicles (AUVs) has improved the feasibility and flexibility of submarine exploration, allowing for the introduction of numerous on-board sensors, enhanced hardware capabilities, and advanced processing tools to improve the entire sensing process. Nevertheless, underwater robot tasks usually require state-of-the-art technology and scientific knowledge due to the tough environmental conditions under which UAV sensing takes place. In general, underwater images suffer from light absorption and scattering effects, which have been extensively reviewed in the literature. Water light absorption is wavelength- and depth-dependent. The red-light component is severely attenuated within the first meters, leading to a rapid incremental dominance of green and blue wavelengths on images. At very high depths, green light is also absorbed and only the blue component is present [1].

Modern AUVs are usually equipped with numerous sensors, depending on the target mission and requirements. The most adopted are two-dimensional sonar imaging, photographic cameras or GPS, alongside field-specific transducers such as pH or oxygen meters [2,3]. Not only have sensor devices been upgraded, but in recent years, UUVs have also benefited from both the recent advancements in machine learning tools and enhanced hardware-processing capabilities. The first aspect catalyzed a processing revolution, as in many other disciplines. With respect to the second aspect, typical, low-cost, open-source CPU-processing boards are extensively used in the literature [4,5] and, due to recent advances in nanoelectronics and artificial intelligence (AI), low-powered GPU boards (mostly employed for artificial neural network solutions) are also starting to be installed in AUVs. Special features such as robot navigation and localization, and constraints such as battery autonomy, sensor space availability and on-board processing power, also need to be considered (see Section 1).

AUVs have a vast variety of applications. These vehicles have been used in the following industries: oil and gas industry, improving the cost and efficiency of offshore projects; underwater acoustics, providing modern sensors to capture and simulate natural environments; chemical industry, working in tough environments; hydroelectric industry, assisting in hydrostructure inspections; water management industry for portable water inspection; offshore and shipping industry; science and research industry; sport sector; television and film production industry; underwater engineering connection solutions; infrastructure inspection; maritime salvage sector; environmental monitoring; plastic production and military industry [6,7].

After analyzing the influence of the special characteristics of the underwater environment, we proceed with the first critical image processing step: underwater image optics and acquisition. This could potentially influence the subsequent processing stages in terms of image quality, computational cost, performance, or algorithm accuracy. These next stages range from preprocessing methods (e.g., white balance, color reconstruction, spatial blurring) to the most advanced algorithm processing (including recent deep ANN image algorithms). Throughout the paper, these stages are studied from the perspective of their performance and impact factors on image processing, as well as the critical issue identification of AUVs.

The remaining sections are devoted to the criteria used to select and classify the most relevant scientific papers on the subject (Section 1), as well as the identification of relevant critical issues in both underwater imaging (Section 2) and their subsequent algorithmic processing (Section 3). Finally, the conclusions are summarized in Section 5.

### 1.1. Paper Selection Criteria

This work followed a thorough literature analysis of underwater imaging and their corresponding algorithm strategies, with a special focus on state-of-the-art techniques from recently published articles. The methodological approach given by the PRISMA 2020 guidelines [8] was tailored and shortened to identify critical issues in this field. Specifically, the PRISMA methods that this section focused on are those of eligibility, information sources, deletion process, the data-collection process, data items, synthesis methods, and certainty assessments. Other aspects of PRISMA, such as search strategy, study risk and reporting of bias assessment, effect measures, and other, similar ones, were discarded due to the limited availability of related papers and the special nature of the proposed objective. The main topics according to the PRISMA results are described below.

In addition, and as a preliminary step, several reviews were explored to acquire a general perspective on diverse underwater vision topics, such as image preprocessing and current algorithmic approaches. The aforementioned strategy allows for this work to further analyze specific underwater vision topics within the imaging workflow to comprehensively detect critical issues and future perspectives in this field.

Among the limited availability of literature reviewsin this area, topics such as underwater optical imaging [9], underwater image processing [10,11], real-time underwater image enhancement [12] and restoration methods [1], as well as deep learning techniques for underwater image classification [13], were relevant topics.

Moreover, current underwater sensing challenges were also considered. For example, some interesting topics include underwater active optical 3D scanners [14], visual–inertial simultaneous localization and mapping [15], the integration of acoustic sensing with the onboard inertial navigation system [16], target detection and recognition in underwater turbid areas [17], and fish geometry measurements from hatchery images [18].

Despite the fact that underwater sensing reviews are currently scarce, some of them are centered on obtaining a general overview of the topic, usually focused on the categorization of optical images [9,10]. Thus, future and critical issues are not usually detected; the most favorable cases of those that were studied briefly mention some general issues in the conclusion that are to be left for future work [19]. On the contrary, the aim of this work is to provide a comprehensive, step-by-step, critical issue analysis and future prospects regarding the entire underwater vision process, considering the present technological and algorithmic state-of-the-art in which we are involved. Furthermore, a novel underwater vision framework is provided based on the inclusion of relevant stages that were raised by the analysis of critical issues, resulting in a generalization of current underwater vision frameworks. This methodology allows for some specific underwater topics and applications, such as camera optics and underwater mosaicking, to be integrated into this workflow.

Due to the limited availability of articles on underwater imaging, the inclusion criteria applied in this work ensure the flexibility needed to accomplish a general analysis of the underwater vision field over a broad range of topics. The main scientific search engines utilized in this work are Google Scholar, ACM, IEEE, FamaUS [20] and MezquitaUCO [21].

To successfully provide critical issues and future perspectives in this field, our inclusion criteria focus on the most recently published articles (ranging from 2016 to 2022, both included), which were the main contributors to the total number of included papers (89 articles, 74.2%). The remaining sum of articles was sampled over a [1990–2015] timespan (31 articles, 25.8%). The total research list comprises 120 articles; however, not all these articles are included in the reference list. Due to the scarcity of published papers in this field, the former sample of historical papers is relevant and could be considered suitable for a general analysis of the underwater vision development.

As for the exclusion criteria, only English-language publications were considered, and military applications, bathymetric unmanned surface vehicles and satellite images were discarded.

**Figure 1:**
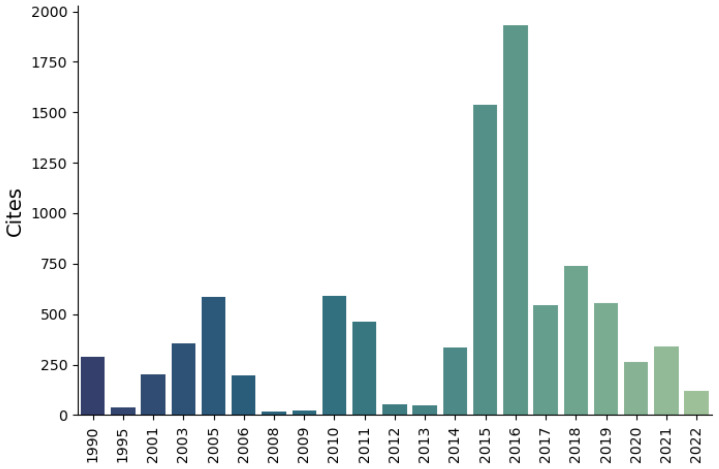
Yearly citations per publication date (paper [23] has been excluded to preserve graph scale).

Figure 1 classifies the 120 research articles according to their publication year, compared to the sum of annual citations. A small subset of these articles was not cited in this work, as they are repetitive topics or do not represent any advancement in the definitive list of detected critical issues. Several specific critical issues have been recurrently cited in recent years due to their relevance. For instance, a 2014 publication successfully tackles a novel topic, retinex theory [22], gathering special attention and leading to great repercussions in this field. Moreover, in 2015 and 2018, very successful articles emerged covering topics such as image-quality evaluation, underwater image restoration, color correction, image dehazing and contrast enhancement, which were shown to have remarkable scientific repercussions.

Furthermore, an evident example occurred between 2016 and 2018, when UUV-related neural networks were solidly applied, resulting in a high reference count being found during the last five-year period. It is worth mentioning that a 2014 image-quality assessment paper [23] was excluded from Figure 1 to preserve graph scale, as it exceeds 40,000 references.

Consequently, some critical topics were demonstrated to be relevant factors for underwater AUV vision, influencing both current and future developments in this field. Hence, our work will focus on critical topics with a remarkable reference count, while also including novel issues that have begun to attract attention in state-of-the-art underwater imaging.

### 1.2. Classification Criteria

**Figure 2:**
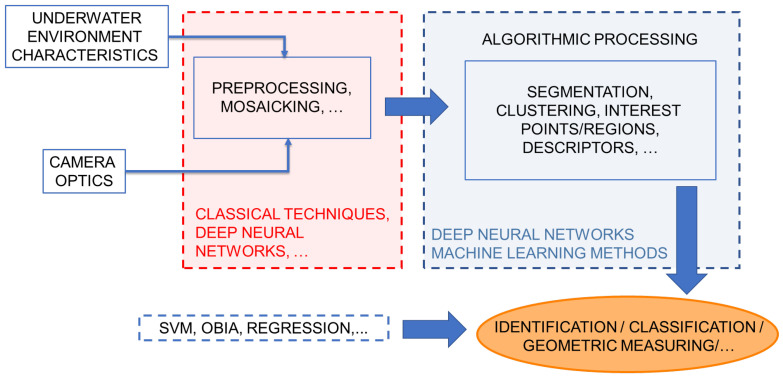
Underwater Imaging Workflow. Red, blue and orange boxes comprise, resp.: examples of algorithms used in the preprocessing stage, main algorithmic processing from low– to high–level programming, and some relevant final tasks.

Due to its general nature, the stated critical issue approach can be sensitive to a broad range of specific applications. In Figure 2, the most common stages of the underwater imaging workflow are generalized and expressed as a flowchart. The proposed critical issue framework integrates the following stages:

1. Underwater environment;
2. Camera optics;
3. Preprocessing;
4. Image mosaicking techniques;
5. Algorithmic processing.

Although the last stage is extensive, it can be divided into two main categories: segmentation and points/regions of interest.

The most common underwater workflow followed in the literature comprises submarine environment modeling, image preprocessing and the main algorithmic stages [24,25,26]. This work further integrates relevant stages, such as camera optics and image mosaicking, to provide a generalized underwater framework while detecting critical issues throughout the process. It is worth noting that image mosaicking is an optional processing stage, which enables orthomosaic production applications to be integrated in this framework, as the following algorithmic processing is generalized and could be applied to further information extraction. Moreover, algorithmic processing has also been categorized in this paper regarding three robotics algorithm stages: segmentation/clustering, points/regions of interest and descriptors. These specific phases lead to the final aim of the underwater workflow, which could be classified as an identification, classification or geometric measuring task.

Although this flow is similar to that of aerial imaging, all stages are deeply influenced by the special features and difficulties that underwater imaging presents, as discussed below. It is worth noting that there is not a solid, robust solution in this field to date; instead, a combination of different approaches are often needed to provide satisfactory results in specific underwater applications and scenarios. However, the proposed workflow can be adapted to a wide range of applications due to its general nature.

**Figure 3:**
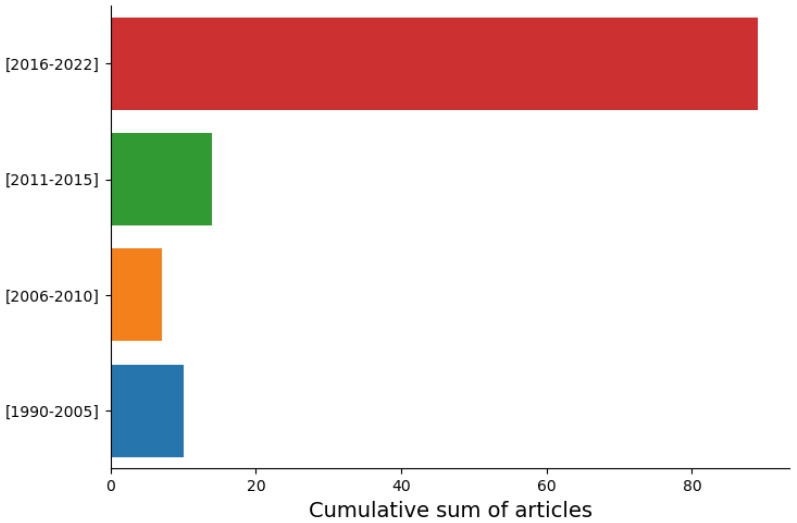
Underwater imaging papers per yearly–interval.

This algorithmic categorization is representative of the latest trends in underwater vision research, as the number of published articles substantially increased from 2016 to 2022 (see Figure 3).

**Figure 4:**
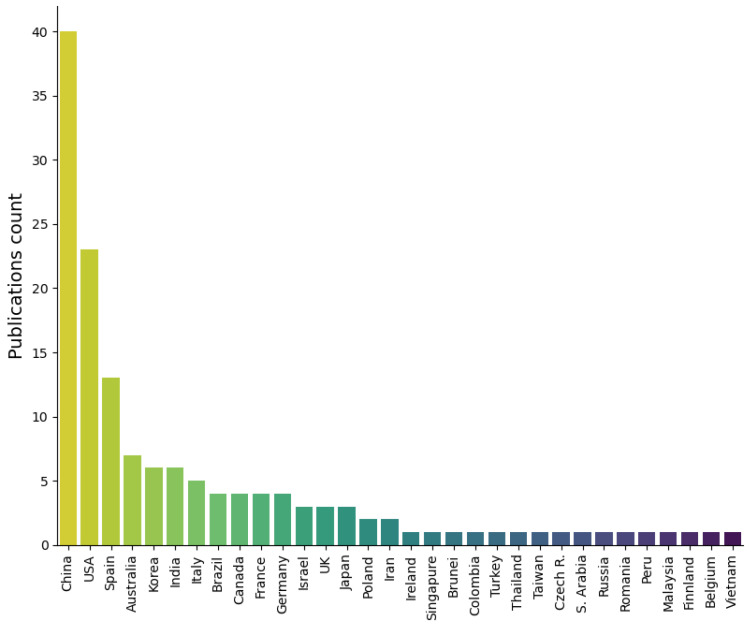
Underwater vision publications by country.

The publications on underwater vision by country are represented in Figure 4. Among the countries with a greater number of underwater imaging publications, we find China, the USA, Spain, Australia, and South Korea. It can be observed that countries with a greater population, sea access and both marine- and fluvial-related industries (such as fish farms or a military) are favored in this chart and tend to investigate this issue more extensively. Furthermore, these statistics reflect the latest rapid evolution in underwater imaging (see Figure 3).

The practice of underwater-image restoration methods can be categorized into two main classes: hardware-based and software-based. Hardware image enhancement includes polarization, range-gated imaging, fluorescence imaging and stereo imaging [10]. Software-based models are oriented toward wavelength compensation and color reconstruction. As a final step, image quality metrics are used to assess the final image outcome [10]. An alternative categorization includes hardware-based laser and multi-imaging techniques, while highlighting the relevance of de-hazing, de-flickering, de-scattering and de-noising models as software-based algorithms [12].

**Figure 5:**
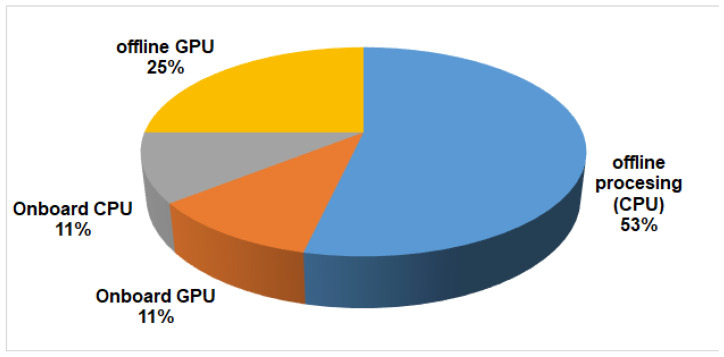
Underwater computing framework strategy.

Figure 5 provides an overview of the computing framework used by the analyzed underwater articles. A first division casts out two main categories: onboard and offline processing. Although these two categories are applied in very different scenarios (for example, avoiding obstacles cannot be implemented offline), offline processing is currently the preferred approach for AUVs (78.6%). Owing to the underwater vision challenges, a higher computational cost could be expected from comprehensive image-preprocessing stages and deep neural network detection algorithms. Offline processing usually enables greater algorithm flexibility and processing power in many applications.

A second classification level discerns between CPU and GPU platforms, with the former being the most extended due to its flexibility. Alternatively, GPU processing is an emerging field, currently promoted by deep convolutional neural networks (CNN), which enables parallel image processing.

Moreover, onboard processing is a recent strategy, made possible by distributed computing platforms, an approach adopted by the 21.4% of the included articles. For instance, low-size and cost CPU and GPU boards are commercially available for installation in both UAVs and UUVs. Despite its limitations, onboard processing is capable of providing feasible solutions in some scenarios, such as image enhancement or basic neural networks with a low number of layers for detection tasks [27,28]. In this case, both CPU and GPU onboard processing platforms are equally represented due to their low sample size, novelty, and low current rate of adoption of this approach.

**Figure 6:**
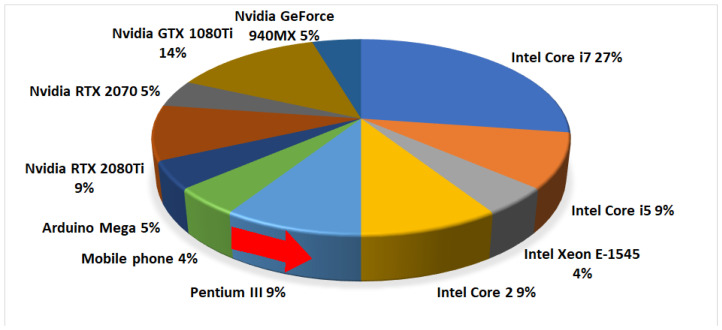
Offline processing in underwater imaging: CPU and GPU platform ratios sorted in chronological order. The first commercial processor is marked with a red arrow.

Figure 6 shows some typical commercial CPU and GPU platforms utilized in underwater offline processing, sorted in chronological order, thus highlighting the computational evolution of underwater vision. High-performance CPUs are the most representative category; however, mobile phones and portable computing seem to be a feasible solution in specific scenarios. Even more recent platforms are being used for GPU processing, evidencing their early incursion in offline processing alongside the incremental adoption of deep CNN image algorithms in recent years.

Regarding onboard processing, some CPU examples include Arduino Mega and NVIDIA Denver 2. Alternatively, GPU platforms such as the NVIDIA GTX 1080Ti and Geforce Pascal series have also been installed on AUVs. Moreover, FPGA-based platforms (Xilinx ZYNQ 7000) are beginning to appear in AUVs for real-time neural network processing, but they are not very a widespread solution at present [28,29]. In some recent cases, both CPU and GPU units can be available in a single board for AI computing, with this being the case of the Jetson TX2 module.

## 2. Critical Issues in AUV Underwater Imaging

**Figure 7:**
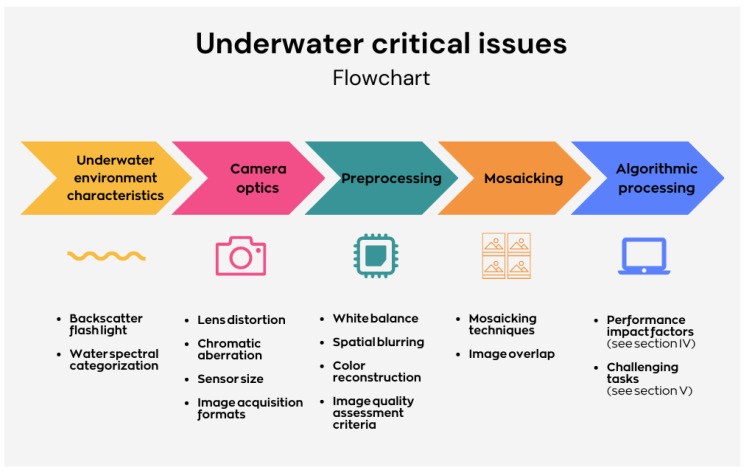
Summary of critical issues in underwater environments.

Following the order given by the previous underwater imaging workflow, we present a guide that integrates the most relevant physical and technical factors detected in the literature, in addition to several influential technical concepts that potentially affect or limit the performance of AUV underwater imaging exploration. This work introduces a novel approach by integrating camera optics and underwater image mosaicking in the same workflow, among other topics, thus helping to unify several underwater fields while drawing attention to both classical and state-of-the-art critical issues. Due to its general nature and broad range of applications, the stated critical issue approach can be sensitive to each specific underwater imaging application. A summary of the current underwater critical issues is presented in Figure 7, which are to be described in the rest of this Section.

More specifically, additional scattering effects result from light diffusion, leading to blurring effects in visible-spectra imaging, which, in turn, produce flickering on shiny days and vignetting when using an artificial light source [10].

As a result, the main underwater light components are direct, forward-scattering and back-scattering, with the latter having a greater impact on image quality. This effect is usually aggravated in practice with the use of artificial light sources, which tend not only to amplify background light reflection, but also highlight small suspended particles within the camera-object region [1].

Scattering components influence underwater photographic imaging in terms of reduced contrast and sharpness, whereas nonuniform spectral absorption leads to a restricted dynamic range and, hence, to a loss in color information [30]. The combination of the above-mentioned effects causes the image quality to be downgraded from its initial capture.

### 2.1. Underwater Environment Characteristics

#### 2.1.1. Backscatter Flash Light

**Figure 8:**
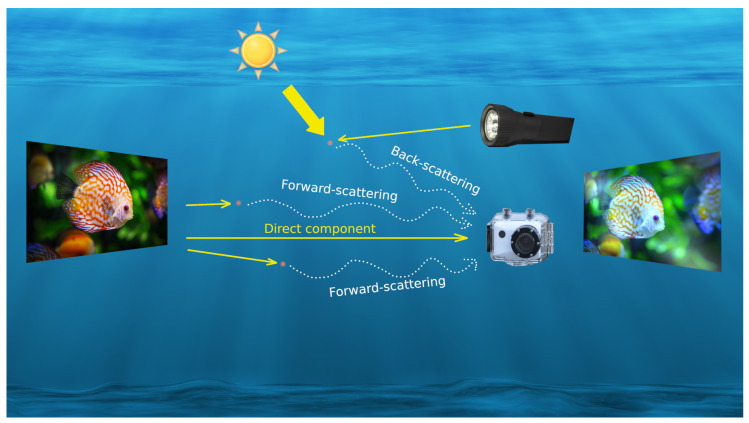
Underwater light components and their influence on AUV imaging. The solid yellow arrow represents the original light font prior to splitting in direct, forward and backscattering, while the latter two are diffuse components represented with dotted arrows. The cumulative effect of scattering and wavelength absorption on underwater images is shown in the right.

Solar radiation faces a change in medium from air to water, thus varying the refraction index according to specific physical and chemical water properties. Light absorption and scattering are the two main underwater issues [31]. Figure 8 illustrates how the presence of suspended underwater particles generates scattering light components. There are two light sources that can introduce scattering problems: solar and artificial. Solar scattering effects are caused by the interaction of light beams with underwater particles, accounting for the general diffuse component related to both forward- and back-scattering effects. Forward-scattering is generally observed when direct light beams reflected by the object interact with particles within the camera’s field of view, thus contributing to image-veiling. However, forward-scattering affects underwater vision to a lesser extent, as most of the diffused light beams are reflected in the opposite direction of the camera. Conversely, back-scattering is mainly generated by artificial flash light sources at short distances, and an over-accentuation of particles within the camera-object region is usually observed. Furthermore, Figure 8 sketches a typical raw image outcome, highlighting both back-scattering (intense light reflections) and wavelength absorption (color bias and information loss) effects on the acquired images. Consequently, back-scattering has been a well-known critical issue with a great impact on image quality. In this section, we discuss possible solutions to both of these effects.

The distance between the camera and the flash is a critical issue that has a great impact on both image back-scattering and quality outcomes. Due to its simplicity and flexibility in terrestrial photography, imaging setups tend to place the flash light near the camera body. Despite being a straightforward solution, this approach markedly constrains image quality from the very beginning. Firstly, the frontal flash displacement with respect to the object further emphasizes back-scattering effects due to the direct illumination of particles within the camera-object region. This is a serious concern, as underwater flash light mostly faces darker backgrounds in deeper water scenarios. Second, proximity to the flash camera is detrimental to contrast transmittance [32], as the main artificial light source adds a frontal, harsh illumination to the scene, restricting the camera’s ability to discern smoother details, and thus reducing the dynamic range. A third relevant aspect accounts for the very low range of lengths in which conventional displacements are capable of providing sufficient illumination. An improved approach is side-lightning [33]. An in-depth study of this setup analyzes the contrast transmittance as a function of the distance of the light source from the camera at different water depths, allowing for a comparison with the traditional displacement strategy, providing further evidence on this subject [32]. In this work, even small increments in the horizontal flash distance position from the camera lead to a greatly enhanced image contrast quality and an improvement in attenuation length range, notably lowering the impact that back-scattering has on the captured image. As a result, horizontal flash-to-camera distances between 3 and 5 m may be the most suitable. Based on the data encountered in [32], we can conclude that this interval could provide a good response for a wide range of water depths of up to 50 m, while further depths would require greater horizontal distances.

Due to the limited dimensions of the UUV, even achieving the proposed side-lightning distance could be challenging. At higher water depths, a proper horizontal distance may not even be feasible. When considering the foremost importance that early quality-preserving stages have, especially in the underwater field, alternative solutions have been practiced in the underwater photography field. Flash light diffusers favor homogeneous illumination and a wider light angle, and also soften shades and edges in the scene [34]. By these means, increased light energy could be available at the periphery of the scene. However, this technique comes with a loss in the length range compared to direct flash light, being only suitable for near-field applications. In shallow waters, underwater photographers tend to work only on the edge of the light beam to reduce back-scattering. In this case scenario, positioning the flash to prevent light diffusion is critical, as a narrower error margin is available. Finally, an additional photographic approach relies on the use of partial flash power settings in search for a compromise between light intensity and low back-scattering that could enhance image quality.

#### 2.1.2. Water Spectral Categorization

The underwater imaging case scenario depends on critical multifactorial issues in the first acquisition stages. As a result, the forthcoming image preprocessing strategy is greatly influenced by the scene. Indeed, water spectral behaviors are manifestly non-uniform, as a broad range of underwater scenarios and water characteristics are possible. Interestingly, the meteorological conditions in the same geographical area may also be influential. In this regard, Jerlov water types are a commonly used means of underwater classification in the literature, which considers radiation absorption and scattering properties to categorize the spectral behavior of water [35]. In this paper, five Jerlov water models are considered for both open ocean and coastal water types. Ocean water types show greater differences in diffuse absorption coefficients as well as a more dominant blue component, whereas in coastal environments, the green channel is predominant. An interesting and solid approach, sometimes followed in underwater image preprocessing, consists of establishing a preliminary Jerlov water-type clustering of underwater imaging datasets prior to the application of image-enhancement algorithms [36].

### 2.2. Camera Optics

Camera optics are of special relevance to capturing the best-quality images possible. Although several papers have studied some optical effects, such as lens distortion and chromatic aberration, the underwater literature covering this topic is currently scarce and centered on specific issues. In this section, we follow an original approach by applying professional photography and physics knowledge to the underwater scientific image field, first incorporating the former, and then additional critical related issues, to the underwater imaging framework.

#### 2.2.1. Lens Distortion

Photographic cameras introduce some light effects during image acquisition. When solar light beams interact with the camera lens, refraction occurs due to a change in medium. Camera lenses are manufactured with standardized diameters to accommodate a variety of scenes and requirements, ranging from 15 mm to 200 mm and beyond. Lens distortion is negligible between 50 and 100 mm, whereas the most extreme lens diameters suffer the greatest distortion. Due to UUVs’ weight, size and economic constraints, most of the popular underwater imaging setups are based on wide-angle camera lenses, a category typically including lenses under a 35 mm diameter. Considering the additional zoom effect that the submarine environment causes in standard camera lenses, smaller lenses are preferred to provide a reasonably wide field of view. In this sense, the fisheye lens is a wide-angle, bottom-end subcategory that is commonly utilized in underwater imaging. Consequently, underwater images captured with ultra-wide angle lens tend to have a strong Barrel distortion. Because of their converging shape, this effect is greatly accentuated towards the edges of the image. Images taken in dark underwater scenarios tend to suffer from a vignetting effect, which alleviates distortion in the most noticeable areas provided that the camera is installed in the focal center of the spherical enclosure [37]. Despite this fact, some applications requiring enhanced geometrical precision for distance and area measurements would benefit from the application of underwater optical calibration strategies. Among these optical issues, lens distortion has been shown to be relevant due to the higher rate of calibration errors [38]. Some authors implemented approaches in an attempt to overcome this issue in aerial imaging. For example, an embedded camera lens distortion correction method for mobile applications, based on a two-stage process, was proposed in [39] to correct both geometric and photometric distortion in low-cost digital cameras. These authors achieved good results with a low computational cost through parallel computing for embedded systems, although the experimental results are heterogeneous depending on the camera model, and additional image preprocessing stages are needed to alleviate these differences. A proposed Barrel distortion correction running on a digital signal processor (DSP) and based on a lookup table method enables users to adjust the correction and compression factors to obtain a corrected image with low edge information loss [40]. An additional correction method for a strong fisheye and wide-angle lens based on 3D space circle fitting has been published [41], yielding robust results with a simple, fast algorithm as a first approximation to more complex methods. Furthermore, lens distortion correction has also been extended to multi-image registration [42].

#### 2.2.2. Chromatic Aberration

Chromatic aberration is a well-known lens effect in professional photography. It is caused by color-wavelength beam deviations as light propagates through the camera lens medium, producing different refraction angles as a function of the input wavelength. The issue is that beam deviations are different from those calculated for a camera lens operating in air because the water refraction index is different from that of air (more exactly, the difference in the refraction index between water and glass is very different from that of air and glass). Chromatic aberration causes some color artifacts in images, becoming most visually noticeable in the objects’ borders and edges. This can be noticed when a homogeneous color background is present. Despite its relevance in professional photography, research on this topic in the underwater imaging field is highly scarce and was absent until recent years. Hence, chromatic aberration is a clear issue in underwater imagery, as a change in the medium can introduce significant chromatic aberrations that affect both achievable precision and accuracy. Several authors have developed chromatic aberration correction algorithms for terrestrial images based on image segmentation [43] and false color filtering for embedded systems, without pre-calibration requirements, designed to correct images at a local scale [44].

A recently published article has exhaustively investigated the influence of chromatic aberration on underwater imagery processing, showing relevant results through an in-depth and well-conducted comparison experiment between four low-cost cameras calibrated in air and underwater [45]. The results show that significant lateral and longitudinal chromatic aberrations can be observed in underwater datasets, while the same in-air calibrated cameras did not show this effect. Both distortion profiles differed by three orders of magnitude, while the root mean square (RMS) calibration values were 3–6 times higher than those in air.

Moreover, some important drawbacks related to chromatic aberration are a diffused, diluted effect on objects’ borders, leading to an edge sharpness loss, as well as local false-color representation. Presumably, this effect may have an impact on some image algorithms, leading to a suboptimal performance.

#### 2.2.3. Sensor Size

Due to AUVs’ weight, space, technical limitations and economic constraints, the vast majority of cameras date in underwater projects to date hae been low-cost, ultra-compact and lightweight, and equipped with small sensors. These pre-installed setups provide some underwater configurations and show a reasonably good performance for everyday situations. However, the underwater environment is challenging and diverse; as a result, these setups are just a first-step approximation to meet specific submarine applications or mission requirements. In this sense, a wider selection of modern cameras has been standardized in the photography field to provide better sensor characteristics for the intended application.

The critical issues discussed in this section have analyzed the relevant optical effects caused by the camera lens. The next step is the photoelectric conversion of the camera sensor. This is a key factor for every photographic camera, allowing for high-quality images to be acquired and delivered. From a material perspective, current commercial cameras implement either CMOS-based sensors or CCD technology, with the former being the most extended. Despite minor differences in terms of light sensitivity and noise, current state-of-the-art sensors achieve roughly the same technical characteristics. Instead, an important critical issue is the sensor size. Digital sensors establish Full-Frame as the main size reference, which has been the traditional 36 × 24 mm film size in small-format analog cameras. Due to the complexity and cost of the digital sensor, smaller sizes have been standardized. Some popular commercial sizes, in descending order, are APS-C (25.1 × 16.7 mm), micro 4/3 (17.3 × 13.0 mm), 1′ (12.8 × 9.6 mm) or ½.55′ (6.17 × 4.55 mm) [46], with the latter two being most commonly utilized in aerial drones and mobile phones, respectively.

Sensor size has not been considered to date in underwater imaging. Nevertheless, its importance is remarkable, especially when dealing with challenging environments such as underwater exploration. In fact, the accuracy of most channel-wise light attenuation models in this field depends on the camera sensitivity and color calibration parameters [47], with the former being an intrinsic camera sensor characteristic. In particular, image quality is related to both camera sensor size and pixel size. As larger sensors tend to incorporate larger pixel sizes, only the sensor dimension is usually considered as a quality feature. Larger pixel cells are more efficient at gathering light, enabling a greater dynamic range, sensitivity, image sharpness, vivid and natural-looking colors and noise performance. Although modern cameras are able to work correctly under good lightning conditions, the previously mentioned factors have become incrementally more relevant as lightning conditions become unfavorable and challenging, such as in underwater scenarios. Furthermore, the camera sensor is not only responsible for better image quality captures but also allows for extended technical flexibility. As an example, a wider depth of field and an ample field-of-view range are available, with the latter being a key requirement in underwater imaging to allow for a general, panoramic scene view to fit in the image.

Although the previously mentioned setup is straightforward to install and use, a compromise between quality and technical flexibility should be achieved from the start of an underwater project.

In sum, it could be highly beneficial to consider the special difficulties faced in underwater environments whenn searching for a camera setup offering a better compromise between cost, size, weight and quality.

#### 2.2.4. Image Acquisition Formats

Some underwater publications briefly mention general aspects of this issue. Some proposals consist of choosing between lossy and lossless compression according to the specific scenario, user needs and transmission quality conditions [48]. Lossless data acquisition is a simpler and faster solution that reduces data transfer overheads for applications with a high number of small-size images, [49]. In the same line, lossless data compression can be efficient for high-resolution, real-time microscopic images such as zooplankton species [50].

The output image file format is a less evaluated topic. Therefore, we extensively analyze the critical issues we believe are worth considering in underwater imaging. This option can be selected in the camera settings as a final acquisition step. Essentially, there are two main image formats that influence further processing stages: lossy (JPEG) and lossless (RAW). JPEG is a well-known lossy compressed image file format that has been a standard since the introduction of digital cameras due to its easy hardware algorithm implementation, low computational cost, lower file size and wide compatibility, providing a high-quality image output for photographic images. Today’s modern cameras are capable of working with very high pixel resolutions, usually well above high-definition standards. For high-resolution images, JPEG compression has a much lower impact on picture quality compared to those lower-resolution standards. However, the actual drawback of lossy image sources is that most of the additional sensor information is discarded during the compression process, leaving very little room for image post-processing operations such as exposure correction [51], image enhancement, color restoration or contrast adjustment. Therefore, the potential results of preprocessing strategies are somewhat limited, as these formats were designed to lead to a final product. On a professional level, when a lossy image workflow is selected, image preprocessing tasks are managed directly on-camera to obtain a close enough final image, reducing the need for further limited off-camera preprocessing operations as possible. It is worth noting that the off-camera preprocessing stages that are applied introduce small, cumulative image quality losses due to lossy image file re-saving.

The preferred professional photography workflow relies on lossless image file formats, commonly called RAW files. These formats capture the complete sensor information, preserving all scene information for professional postprocessing purposes prior to a final export to JPEG or other lossy formats. In this case, a proper image-preprocessing stage can be developed, taking full advantage of the camera’s sensor abilities. For instance, recurrent image operations such as histogram sliding, contrast enhancement, or color restoration could be optimally utilized. Considering the tough underwater environment characteristics and the necessity for a comprehensive image preprocessing stage to restore scene information, this approach provides higher quality and flexibility, and increased tolerance to upstream, unplanned acquisition mistakes in underwater imaging.

Finally, a prominent underwater imaging alternative is based on video capture. Most modern cameras are capable of providing both image and video-recording features. In underwater projects, both approaches have been utilized. Some advantages of video sources are automatic image stream throughput, visual-inertial navigation system adequacy, and the availability of frame rate options. On the contrary, video sources demand additional computing stages such as video-to-image frame conversion and increased computational cost through video codec computations. In fact, both the image- and video-encoding processes are an issue in deep-sea exploration [52] due to the limitations of underwater communication. Despite the fact that only lossy video codecs are available in commercial cameras, there are video formats with a greater quality output. By default, very high bitrate and high-resolution video streams are enabled for tasks such as underwater imaging, providing a fairly adequate, flexible solution.

Consequently, a post-processing quality compromise should be considered. The most unfavorable of the studied cases is on-board processing, as seen in Figure 5, which was selected by 21% of the related articles. Even though the on-board computational cost currently limits very high-resolution video streams, some high-quality and low-compression video formats may be suitable in cases where enough data storage and a sufficient processing time are available for the intended application.

### 2.3. Preprocessing

#### 2.3.1. White Balance

An extensively applied software-based image preprocessing technique in photography is white balance. The aim of this method is to obtain an unbiased color representation of captured images, independently of specific, unusual lightning scene conditions. More precisely, a common procedure is: the mean global RGB color value is first calculated, and then the correction factor for each independent color channel is computed by normalizing the mean RGB value to each of the three independent red, green and blue contributions, respectively. This procedure compensates for dominant color imbalances due to non-ideal light sources with respect to the visible spectrum of sunlight. Color temperature is the measurement unit of white balance, allowing for a general, reproducible light-source categorization. Image color information is considered a key attribute in many image-recognition algorithms. Therefore, a truthful and reliable color representation is a critical issue and an active research area in underwater imaging.

White balance is also a necessary and widely applied technique in terrestrial photography. Due to the adverse underwater imaging conditions, notable benefits could be expected from this approach. Considering the blue and green color biases and the additional red channel degradation, limited contrast and blurring are issues of a different nature, and they are usually restored in further preprocessing stages. In this regard, some authors have proposed diverse underwater image enhancement algorithms: a color balance via two-image fusion to promote edge and color contrast is transferred to the recovered image, being reasonably independent of camera settings [53], as well as an alternative approach based on unsharp masking and contrast-limited histogram equalization [54] are some relevant examples. Furthermore, a recent paper has proposed an underwater image-enhancement algorithm based on a different color balance approach via the minimal color loss principle and local adaptive contrast enhancement [55], achieving advantageous results in terms of fast processing, image segmentation, keypoint and saliency detection, although its performance when dealing with low-light underwater images was lacking.

#### 2.3.2. Spatial Blurring

**Figure 9:**
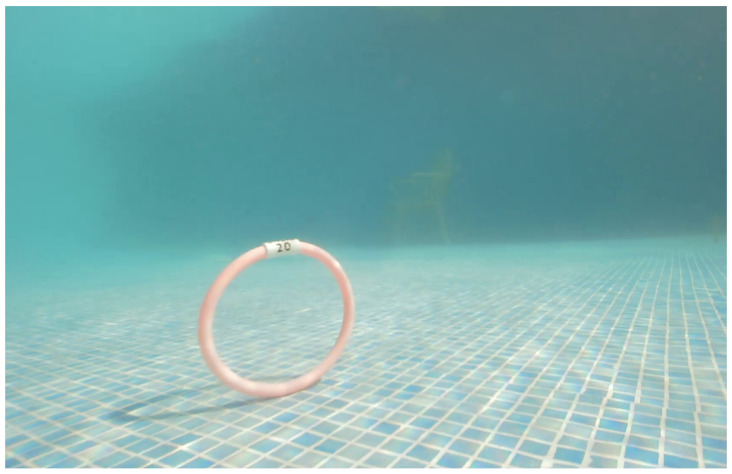
Original video frame captured by our underwater drone in a swimming pool test. The spatial blurring effect and lightning conditions make it difficult to discern the yellow chair in the background.

White balance techniques, as well as some of the derived color balance alternatives, are recurrent approaches in the underwater literature, as they are able to restore underwater images to some extent. However, firstly, poor underwater visibility is a multifactorial issue, and secondly, spatial blurring is an additional remarkable critical issue. As stated previously, backscattering is the main contributor to image contrast degradation, generating a spatially varying veiling light effect that increases at further distances, and typically reaches a maximum cumulative effect in the image background (Figure 9). An additional factor contributing to spatial blurring is the artificial light power decay, which progressively constricts the dynamic range at further distances. A polarization-based approach has been developed for underwater image enhancement [37], considering both white balance and the spatial veiling light effect under natural light sources, which practically doubles the underwater visibility range. Despite the few studies regarding underwater video enhancement, we include, as an example, a submarine video dehazing algorithm [56] based on dark channel prior and time-domain information fusion.

#### 2.3.3. Color Reconstruction

Wavelength attenuation in the visible spectrum is a notorious underwater effect that limits vision quality and potential, causing a strong color information imbalance, which is most detrimental to the red channel. The combined effect of backscattering and light attenuation results in a downgrade in image contrast and loss of chrominance. Essentially, color is a basic attribute for many image algorithms and artificial intelligence approaches. Therefore, color reconstruction is an active research area as well as a critical underwater issue. Several image color restoration algorithms have been developed in order to recover scene information to the greatest possible extent possible. Owing to the complexity of underwater image restoration and enhancement procedures, some sophisticated approaches, such as model-based enhancement and deep learning, have been developed in the literature. Bioinspired human color perception algorithms have been applied in underwater imaging to enhance local image contrast, with the multiscale retinex being one of the most extended [57], and known as the first work or the retinex theory-based contrast enhancement method. It offers a good trade-off between performance and dynamic range compression, as several authors have asserted [22]. This method has two versions: color balance restoration for each single RGB channel or luminance channel correction, with the former being the most suitable for underwater vision. Additionally, a more efficient and versatile retinex approach, suitable for underwater video enhancement [5], was studied for five types of degraded submarine images based on green, blue, darkness and turbidity attributes, obtaining satisfactory results in the first two categories. Furthermore, an automatic red channel image restoration algorithm [58] has been able to deliver good-quality images and a natural color correction outcome even under artificial light.

Moreover, turbid areas are among the most challenging underwater vision scenarios. An integrated two-step approach based on color restoration and image enhancement for turbid images offers a consistent performance in a variety of shallow-water scenarios [59]. An alternative hybrid restoration approach [60] initially discerns between foreground and background restoration, and then utilizes traditional threshold and masking photogrammetry techniques for the former and a generative adversarial network for the latter.

In recent years, deep convolutional neural networks have been a trend in underwater vision enhancement and super-resolution conversion, with promising results.

Deep CNN methods have been enhanced for underwater haze removal and color correction using a pixel-disrupting strategy. They also allow for improved feature extraction, achieving a very good performance [61]. An alternative CNN approach generates transmission and ambient light estimation maps to learn underwater effects, preserving image details accurately by cross-layer connection and multi-scale estimation [62]. Recently, a CNN-based underwater image enhancement has explored RGB and HSV color spaces to recover luminance and saturation information [63], providing good image detail.

#### 2.3.4. Image Quality Assessment Criteria

Underwater image-quality metrics are currently an active research field as they provide important references for image-quality evaluation, algorithm comparison and feedback for further improvement. Thus, defining assessment criteria is a critical issue that may have an impact on future underwater vision development. The most relevant image-quality metrics usually applied in underwater vision are (a) the structural similarity image netric (SSIM), which considers the human visual ability for structural information detection as a relevant quality factor [23]; (b) underwater-based image-quality metric (UIQM), which includes contrast, sharpness and colorfulness as weighted linear parameters in descending order of importance [64], (c) an underwater color image-quality evaluation metric (UCIQUE) based on a linear combination of chroma deviation, luminance contrast and average saturation in commission internationale de l’éclairage L*a*b (CIELAB) color space, which are ordered in descending degree of importance [65].

Despite the fact that actual image metrics consider relevant characteristics such as colorfulness, luminance, sharpness and contrast, future underwater image-quality metrics may need to include additional factors such as low-level detail preservation or image fidelity. Moreover, some current assumptions in underwater metrics may be inaccurate. As an example, UIQM has the disadvantage of not promoting natural-looking images, especially under turbid scenarios on highly degraded images [59]. To ensure a high-quality image and fidelity outcome after preprocessing, the more suitable filters are based on moderate changes in contrast, sharpness and color, because they prevent excessive saturation, detail and overall information loss with respect to the original image capture. Consequently, future image-quality metrics should consider a better balance between images that natural-looking and highly detailed at the pixel level and their counterparts to produce sharp, vividly contrasted images.

To address this issue, some initial proposals are being published in terrestrial imaging, such as the patch-based contrast quality index (PCQI), which differs from previous models in its ability to build a local contrast quality map and predict local quality variations over space [66]. Future metrics considering this issue could be more successful in assessing contrast detail preservation. We consider approaches in this direction to be a good complement to actual underwater metrics, as they have been included in some articles on image enhancement [53,67]. Regarding algorithm implementation, achieving underwater vision enhancement while preserving fine, low-level details in the scene is a major challenge. A multi-camera super-resolution approach to enhancing underwater video streams [68] is a good example regarding detail preservation. Recently, CNN-based algorithms have offered very good results in this regard [62]. In this sense, the CNN approach has been recently applied to map in-air conditions to artificially degraded underwater images with a focus on local detail retention [69]. We conclude that the definition of PCQI and other quality indexes should be considered, so that future researchers could benefit from a more exhaustive image quality assessment.

### 2.4. Image Overlap and Mosaicking Techniques

Underwater mosaicking is an ongoing research field aiming to inspect the floor surface of lakes, reservoirs and marine environments by obtaining a final map composed of overlapped image tile units. In the case of seafloor mosaicking, further device challenges are met in terms of battery capacity, artificial light power and range. Another set of issues arises from the difficult exploration of uneven seafloor surfaces at considerable depths. Traditionally, underwater image mosaicking has mainly been applied to vision-based navigation, although large mosaic building has been a relevant application since the beginning [70].

At present, underwater image-blending procedures commonly assume low forward and side overlaps, delivering 2D mosaics. It is generally assumed that the interframe motion is mainly translational. However, it is well-known that a prealignment process is needed to ensure a high-quality mosaic outcome when rotations are greater than 10–15% [70]. Although 3D reconstruction benefits from composite image translations on uneven surfaces, the application of this technique for very low overlap scans is not always feasible. Low overlap images are a common practice in underwater mosaicking, mainly due to power constraints [70], although they allow for greater distance coverage as well as data storage reductions [71]. Specifically, the underwater overlap ratios that stated in the literature vary between 15–25% [72] and 35% [71].

Despite being a little developed field, several authors have implemented blending algorithms. For instance, fast underwater image mosaicking proposes an efficient, modified agglomerative hierarchical clustering method to build submaps based on similarity information from feature descriptors, increasing efficiency while reducing image-matching attempts [73]. Furthermore, an efficient feature-based image mosaicking (FIM) method has utilized multiple underwater robots for the topology estimation process, delivering an accurate map [74]. Finally, a recent underwater image blending algorithm based on globally optimal local homographies has been applied to seafloor mosaicking [75]. It was shown that the adoption of a local warp model improved the alignment and minimized local distortions compared to global approaches. The authors also proposed a three-step seafloor mosaicking pipeline consisting of (a) image keypoint extraction and matching, (b) camera orientation estimation, and (c) the fusion of both previous stages, providing a natural-looking mosaic [75].

## 3. Algorithmic Processing

The forthcoming stages of the workflow presented in Section 1.2 comprise the algorithmic processing. As this stage is very wide, we begin with a classification centered on underwater image algorithms in the next subsection. Having established the taxonomy, the rest of the subsections focus on the performance impact factors and the most relevant challenging tasks that were accordingly identified in this field.

### 3.1. Algorithm Classification

A wide variety of processing and identification techniques have been developed as a response to numerous underwater vision applications. Underwater vision typically applies the same general algorithmic abstraction as that of aerial imaging, thus enabling the latter to be adapted by introducing some required changes to this field. Two main types of algorithms can be distinguished: classical image processing (pixel-based manipulation, photogrammetry, or digital elevation modeling, for example) and machine learning algorithms. Even though this variety challenges the determination of an exact classification, the majority of these techniques can be categorized into five main modalities:

(a) Classical imaging techniques;
(b) Machine learning: artificial neural networks and unsupervised classification;
(c) Machine learning: regression;
(d) Support vector machines (SVM);
(e) Object-based image analysis (OBIA).

Classical imaging encompasses a broad variety of techniques regarding image filtering, pixel-based manipulation, color-space representation, photogrammetry, model-based image enhancement, morphology, topological parameters, etc. These algorithms rely on nonlearning methods and are generally well-known and robust, especially for traditional algorithms.

A relevant, classical imaging subcategory is photogrammetry. These techniques are regarded as a first step to generate orthomosaics or digital elevation models (DEM). It encompasses precise scene reconstruction from several superimposed images captured by one or several sensors, establishing the geometric properties of these two-dimensional images to obtain a 2D or even 3D mosaic provided that images captured from different points of view were available.

ANNs are among the most utilized machine learning methods at present. Within this category, CNNs were demonstrated to be effective for object detection and classification in large datasets. Conversely, these algorithms tend to have higher computational cost and demanding training datasets (see Section 3.3). In this sense, post-processing or external informatics infrastructures (cloud computing) may additionally be needed. Furthermore, unsupervised classification encompasses machine learning fields such as clustering (KNN, hierarchical or probabilistic clustering), data compression (PCA, singular value decomposition or SVD) or generative models. It is worth noting that neural networks can be designed to work with both supervised and unsupervised datasets.

Regression models are not only a highly regarded statistic inference method but also a supervised machine learning algorithm. They find an existing relationship among variables by adjusting the coefficients of a target parametric function. These algorithms were extensively applied in several underwater imaging applications, such as image enhancement and preprocessing, image mosaicking construction, and subject geometric measuring algorithms. Moreover, some popular algorithms include multiple linear regression and nonlinear regression, as well as advanced multispectral analysis techniques with a low sample number (sparse regression methods), which aim to extract the basic components from every physical camera pixel.

SVMs are regarded as a nonparametric, statistical learning method and were recently used in various image processing applications. The aim of this algorithm is binary classification through the definition of an optimal hyperplane, enabling a maximum gap margin between two groups. Regarding nonlinear classification, SVM uses several kernel types, which convert nonlinear boundaries to hyperplanes of higher dimensions until the optimal hyperplane is found. In underwater imaging, hyperspectral data provide hundreds of data channels with high feature variability, as well as complex characteristics and nonlinear relations among the spectral bands that could be exploited.

Object-based image analysis (OBIA) is a common learning method that discerns present objects in underwater images. Conversely to pixel-based methods, OBIA elemental analysis units are not pixels, but adjacent pixel groups (objects) with homogeneous spectral values, and hence are considered as a topological entity. This technique can analyze and classify images, exploiting not only individual pixel information, but also geometric, spectral, textural, and, by extension, topological information.

**Figure 10:**
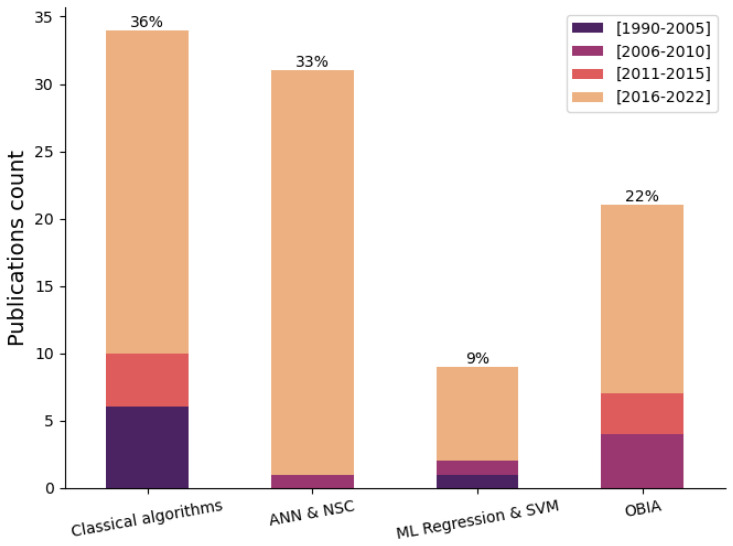
Yearly evolution of underwater image processing algorithms.

As an example, Figure 10 shows the resulting classification of 85 articles related to underwater image algorithms, regardless of the processing stage in which these methods were utilized. Results show that both classical algorithms and artificial neural networks (ANN) and unsupervised classification categories are the two most popular approaches in recent years, representing 69% of the studied articles, while object-based image analysis (OBIA) methods are becoming increasingly relevant due to their potential in superpixel and topological information extraction, forming 22% the total number articles. As a matter of fact, some OBIA concepts are being applied in recent object-based deep neural networks, leading to the promotion of the neural network category. Finally, regression and SVM form the smallest group, with 9% of the total papers. Despite the regression not being represented as the main algorithmic strategy in many articles, it is found in multiple underwater image stages, such as camera optics, preprocessing, and image mosaicking.

Classical algorithms are the most represented category, closely followed by the ANN and NSC groups, as they were demonstrated to perform well in various underwater scenarios, yielding promising results. Furthermore, both categories are usually utilized within the same workflow due to their general applicability, with classical algorithms usually being preferred for optical distortion correction, as well as some image restoration algorithms, prior to the training stage of deep neural networks. As an example of a shared application, both groups are currently being applied to underwater image-enhancement tasks.

Further discussion of these categories, their performance impact factors, and the most relevant high-level tasks that comprise the state-of-art can be found in the following subsections. In summary, and after having analyzed the algorithm categories, we contrasted both the advantages and drawbacks of each technique, leading to the next summary of which methods represent the state-of-art in the field of underwater algorithmic processing: deep neural networks and regression have mostly been used in image preprocessing and mosaicking [76], respectively, whereas SVM and OBIA have served as final identification and classification algorithms. SVM is currently utilized in conjunction with neural networks rather than alone, as the latter tends to obtain a better performance [77,78,79,80], and the combined approach can lead to enhanced algorithms [81,82]. Moreover, regression methods have yielded satisfactory results in geometric measurement tasks, such as fish weight and size measurements in underwater hatchery images [18].

### 3.2. Performance Impact Factors

At present, lossy image formats are among the most abundant image sources, as they allow for important bandwidth reductions and versatility requirements for general-purpose applications. JPEG compression works under the assumption that high-frequency information does not contribute much to the general psycho-visual scene. Although compressed images have been extensively applied as input data to algorithms, very few assessments of their potential impact on algorithmic performance have been published. This is even more notable for underwater imaging.

Classic studies regarding lossy image compression effects on classification tasks support evidence that high-quality classifications could generally be obtained for JPEG compression ratios approaching 10:1 or higher, especially for spatial pattern detection applications [83]. Regarding generic deep learning and neural networks, several studies have assessed the impact of image quality on deep neural networks. Some authors have analyzed this issue under various distortions [84]. In descending order of importance, blur was found to be the most relevant, transversely affecting all four CNNs evaluated in this paper, even at moderate levels; noise is especially relevant for CNNs, with a low number of convolutional layers; JPEG affects neural network performance for compression ratios higher than 10:1 and 30:1 (for JPEG and JPEG 2000, respectively). Finally, contrast affected all four of the studied CNNs, although to a lesser extent compared to previous distortions.

In addition, training on the compressed dataset could raise the training accuracy, but it would not increase the pixel-matching in the original classification. This is a well-known consequence of the elimination of much of the pixel-to-pixel detail that high compression ratios produce [83]. Alternatively, a recent study evaluated the impact of JPEG compression on some common deep learning tasks, such as classification, detection and semantic segmentation [85], finding a steep, significant performance loss from high (10%) to moderate (50%) compression settings.

The influence of segmentation algorithms has been analyzed in many imaging fields. Overall, many studies conclude, for terrestrial images, that an assessment of color orthophotos over lossless and JPEG 2000 compressed images can be established in a few thematic categories, such as dense vegetation, herbaceous, bare lands, road and asphalt, or buildings. The results [86] show that the loss of accuracy at the first compression level was more significant in highly fragmented areas. Consequently, one can extend these classical results to underwater imaging, so that topological segmentation tasks could have a higher sensitivity to lossy compression, as these algorithms tend to work best with pixel-to-pixel detail. This may require either the application of stricter, lower compression thresholds to mitigate quality loss compared to other algorithms or, ideally, a lossless image compression workflow.

Specifically, Ref. [87] analyzed the influence of range, image resolution and compression on underwater stereo video through both high-definition and broadcast resolution video cameras, stating that HD binocular video is capable of measuring objects more accurately over greater ranges, which also facilitates fish taxonomic identification. For photogrammetric applications, moderate compression levels have little influence on the accuracy and precision of measurement tasks, whereas degradation follows a linear progression with compression [88]. This paper also recommends recording video streams in progressive mode, thus avoiding possible interlaced motion artifacts due to the water medium.

Other factors that affect the correct object shape, target recognition, and influence of color on detection performance are detailed in the following lines. A recently published signal transmission error model based on Fourier descriptors, HSV color space and gray-level segmentation [24] conducted preliminary research regarding the influence of object shape and color under various laboratory scenarios (air, shallow water, 40 cm and 80 cm underwater, with and without flow interference and non-uniform lighting effects). This algorithm revealed that green and blue rounded shapes are harder to detect, especially at high depths under non-uniform light. In contrast, shapes with defined edges were accurately detected in nearly all scenarios while providing enhanced robustness to color influence. Moreover, rounded targets were shown to demand higher requirements on interest point algorithms to obtain a smoother segmentation, thus avoiding confusion with polygonal shapes.

### 3.3. Datasets on Underwater Imaging

Even though numerous underwater image restoration methods have been published, underwater image datasets are still limited. Therefore, dataset augmentation methods have become a recurrent strategy to ensure the establishment of the greater image databases that are usually required to train deep neural network algorithms. For instance, a 3D-modeling dataset augmentation method was proposed for AUV real-time operations [89], increasing the significance of rare underwater objects for detection algorithms. Furthermore, some simulated underwater physical effects were included in a synthetic dataset to build an underwater image-enhancement algorithm for infrastructure inspection [90]. A new quantitative underwater dataset was provided to the field, which includes far-field objects at different distances between cameras and objects of several meters of magnitude [91].

However, actual underwater image databases also suffer from qualitative issues, as most of them only provide near-field targets that must be recognized. While foreground targets can be acquired and detected with greater success and simpler enhancement techniques, such as contrast stretching, future image datasets should provide enhanced quantitative and qualitative data by adding the spatial distance variable [19] so that issue-related character and far-field detection could be better addressed.

## 4. Relevant Challenging Tasks

**Figure 11:**
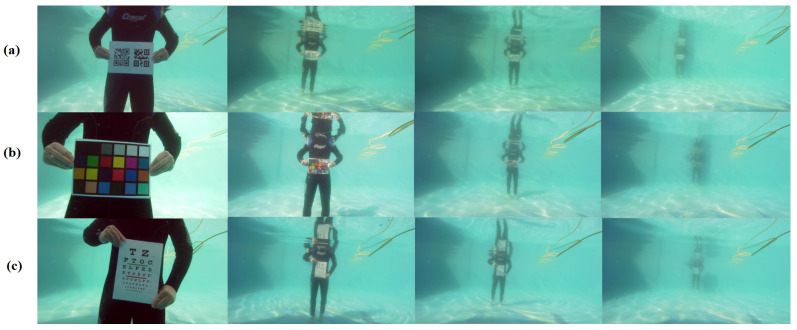
A set of images captured by our underwater drone considering the spatial variables under three different calibration tests: (**a**) QR code patterns, (**b**) a color calibration card common in image processing, and (**c**) text symbols. This provides an example of pattern and color recognition as current challenging tasks. The distances between underwater robot and target from left to right are approximately 1 m, 3 m, 5 m and 7 m, respectively.

Underwater vision applications provide advantageous results as well as wider possibilities in near-field sensing with respect to sonar imaging. However, some important challenges are currently being confronted in underwater imaging so that it can be used near its application limits, as illustrated in Figure 11. In this sense, a key factor is far-field object detection. Binocular vision is a hardware-based approach that provides increased depth perception, enhanced visibility, light sensitivity and better 3D reconstruction capabilities. In our days, underwater far-field imaging is an emerging field. Nevertheless, some initial proposals can be found, such as a stereo-matching algorithm for 3D information recovery [92]. This technique, based on a three-step pyramid resolution approach with anisotropic diffusion, provides better accuracy than the classical block- and semiglobal-matching methods. The choice of similarity measures such as the ZNCC score, alongside census-tranform-based matching and the smoothness transition analysis at the pixel level, provide robustness to light variations, thus approximating the underwater vision requirements. Another method based on superpixel segmentation and polynomial regression [26] has reduced the stereo mismatch for 3D target reconstruction under swimming pool test conditions. In fact, the proposed measurement method can be adequate for camera-object distances of up to 2 m, where the mean error slightly exceeds 5%.

As stated above, future work could focus on more advanced camera calibration strategies such as the pinax model [93] or a generalization of the pinhole model. In this regard, an advanced camera calibration model for underwater 3D scanners has combined the accuracy potential of ray-based modeling with the common principles of pinhole models to provide enhanced precision for geometric measurement tasks [94]. To ensure a simple and flexible calibration process, novel input variables consider air-calibration data and distortion function, glass-refraction index, glass thickness, and the distance between the camera and glass. Simulation results have demonstrated that pinhole modeling can be used for larger object distances without remarkable accuracy losses. However, certain conditions should be satisfied before using the pinhole model at shorter distances.

The Pearson correlation coefficient between the negative logarithm of the estimated transmission map and the true distance has been used to assess spatial visibility. Consequently, far-field evaluation evidences greater differences among existing image-enhancement methods, which generally have low far-field visibility or color bias. These critical issues were previously identified in Section 2.3.2 and Section 2.3.4, respectively.

Feature-extractors are a step in navigation algorithms. In underwater imaging, this task becomes increasingly difficult due to the non-uniform lightning and low visibility [95]. For instance, a real-time stereovision framework [96] has been proposed for AUV navigation. Distance measures to foreground objects are gathered from background substraction techniques. Furthermore, some initial proposals regarding scene-change detection and environment monitoring, oriented toward navigation [97], are being developed.

Moreover, the influence of color on detection performance may not be neglected. As an example, an image metric evaluates the color-correction accuracy through the average angular reproduction error between grayscale patches and pure gray color in RGB space [91] while mitigating the influence of brightness on color representation. This method may help to identify well-balanced image restoration methods that tend to better preserve background information for future detection tasks.

## 5. Conclusions

To contribute to the necessary development of the underwater imaging field, focusing on existing challenges, this work provides a comprehensive, step-by-step critical issue analysis of the entire underwater vision process, considering both the present technological/algorithmic state-of-the-art and the future prospects. This approach evaluated the environmental, optical, image enhancement and algorithmic processing stages to obtain further insights into the first critical image acquisition steps, which could potentially influence further processing stages, while detecting synergies along the imaging workflow. Furthermore, a generalization of the underwater vision framework is provided based on the inclusion of relevant stages raised by the critical issue analysis.

To mitigate underwater back-scattering effects, side-lighting provides better subject definition and textures and, hence, can reduce computational costs in image preprocessing. Furthermore, environmental factors such as water depth, turbidity and flow speed have a great impact on underwater imaging, raising physical and technical challenges that should be considered and evaluated in underwater projects.

The camera optics stage has shown to be a relevant issue; however, this underestimated in the underwater field. Despite the research on terrestrial imaging, no method yields robust results for submarine images. It may seem that a variation in these aerial techniques could work properly on underwater images, but the scientific community should make an effort to overcome this issue. To date, there few camera lens optics have been purposely built for native underwater operation, nor do actual camera lenses consider the optical design parameters for underwater applications. Future work could focus on more advanced camera calibration strategies, such as the pinax model or the further generalization of pinhole models considering some advanced camera optic properties.

Moreover, chromatic aberration has been shown to be a general, transversal critical issue affecting underwater imaging; thus, it could be good practice to evaluate and compensate for such effects in oncoming underwater projects. At the technical level, the selection of higher camera sensor standards may provide advantageous results in terms of image quality, dynamic range, noise performance and light sensitivity considering the challenges and diversity of underwater scenarios.

With respect to underwater image quality assessment, real submarine image datasets are scarce. This is a reason for the popularity of data augmentation strategies in this field, which, in turn, reduce the ability to determine the consistency and adequacy of these metrics. Pixel-level detail preservation and image natural color fidelity should be considered alongside current quality assumptions, so that future researchers could benefit from a more complete and accurate image quality assessment.

One aspect of the algorithmic processing challenges that is worth investigating is how to improve the detection performance of green and blue rounded shapes through more efficient computing strategies. Moreover, several publications have shown that lossy image sources such as JPEG affect segmentation algorithms to a greater extent, whereas classification and neural network algorithms seem to be more robust to lossy compression.

With respect to high-level tasks, the more challenging ones involve far-field imaging, 3D reconstruction and underwater video enhancement. Photographic cameras provide advantageous results in near-field underwater imaging, while sonar applications are preferred for long distances. However, camera imaging is facing challenges regarding uses near its far-field application limits; the aim is to improve its usable range and potential. Binocular vision is a promising approach that provides increased depth perception, visibility and light sensitivity for measurement and 3D reconstruction algorithms. Furthermore, far-field visibility metrics demonstrate some of the shortcomings of current image enhancement methods, which generally tend to have a low spatial visibility or color bias, with both affecting detection performance. Finally, underwater video enhancement is an area that researchers need to further elaborate in the future.

In sum, underwater sensing is a challenging field, influenced by a wide variety of optical and physical effects, sensor constraints, hardware limitations, and a lack of consistency in environmental characteristics. Some strategies that are applied in the first stages can notably reduce the downstream computational costs while potentially providing a better quality outcome. The present critical issue analysis highlights the importance of considering these issues as possible limiting factors, allowing for future researchers in this field to specifically detect and select the most concerning effects.

## Data Availability

The data utilized to extract statistics, as well as the python script for the corresponding figures is available at: https://github.com/mjmoron/Underwater_critical_issues, accessed on 29 March 2023.

## Abbreviations

The following abbreviations are used in this manuscript:

UUV	Unmanned Underwater Vehicle
AUV	Autonomous Underwater Vehicle
ANN	Artificial Neural Network
DSP	Digital Signal Processing
CCD	Charge-Coupled Device
CNN	Convolutional Neural Network
HSV	Hue, Saturation, Value
SSIM	Structural Similarity Index Metric
UIQM	Underwater Image Quality Metric
UCIQUE	Underwater Color Image Quality Evaluation Metric
CIELAB	Commission internationale de l’éclairage L*a*b
PCQI	Patch-based Contrast Quality Index
FIM	Feature-based Image Mosaicking
DEM	Digital Elevation Model
KNN	K-Nearest Neighbours
PCA	Principal Components Analysis
SVD	Singular Value Decomposition
SVM	Support Vector Machine
OBIA	Object-Based Image Analysis
NSC	Non-Supervised Classsification
